# Endoscopic gastric plication complications: early hemorrhagic event and late suture dehiscence. A two-case series

**DOI:** 10.1093/jscr/rjag584

**Published:** 2026-07-10

**Authors:** Maximo V Torres Guaicha, Alberto C Gordillo Calero, Tabata L Tinoco Ortiz, Paul M Tovar-Cobos

**Affiliations:** Department of Surgery, Hospital Metropolitano de Quito, Avenida Mariana de Jesús S/N y Nicolás Arteta, 170521 Quito, Ecuador; Department of Surgery, Hospital Metropolitano de Quito, Avenida Mariana de Jesús S/N y Nicolás Arteta, 170521 Quito, Ecuador; Department of Surgery, Hospital Metropolitano de Quito, Avenida Mariana de Jesús S/N y Nicolás Arteta, 170521 Quito, Ecuador; School of Medicine, Universidad San Francisco de Quito, Avenida Diego de Robles S/N y Vía Interoceánica, 170901 Quito, Ecuador

**Keywords:** endoscopic sleeve gastroplasty, suture dehiscence, hemoperitoneum, weight regain, revisional bariatric surgery, suboptimal weight loss

## Abstract

Endoscopic gastric plication (EGP), also known as endoscopic sleeve gastroplasty, is a minimally invasive bariatric procedure that reduces gastric volume through full thickness endoluminal suturing. It has demonstrated aiding weight loss and improvement in obesity-related comorbidities. It is considered safe, with low rates of severe adverse events, but early and long-term complications remain insufficiently characterized. We report two cases of EGP-related complications. The first case involves a 31-year-old woman who presented 2 years after the procedure with persistent dyspepsia and recurrent gain due to suture dehiscence, requiring conversion to Roux-en-Y gastric bypass with removal of gastric remanent. The second case describes a 24-year-old woman who developed an early postoperative hemorrhagic complication secondary to gastroepiploic vessel injury, requiring urgent surgical intervention. These cases illustrate potential EGP severe complications, ranging from early acute life-threatening events to late structural failure, emphasizing the importance of high suspicion, timely diagnosis and individualized management strategies.

## Introduction

Endoscopic gastric plication (EGP) aka endoscopic sleeve gastrectomy is an endoluminal procedure that reduces gastric volume through full-thickness suturing or tissue anchor plications. It has demonstrated effectiveness at promoting weight loss, with meta-analysis showing a mean reduction between 15% and 20% across studies [1]. It also has been shown to be effective at improving diverse comorbidities associated with overweight and obesity, such as hypertension, diabetes, gastroesophageal reflux disease (GERD), Metabolic dysfunction–Associated Steatotic Liver Disease (MASLD), and obstructive sleep apnea (OSA) [2–4].

Because it is an endoscopic procedure EGP offers a less invasive alternative than surgical bariatric interventions and thus is expected to have fewer complications. A meta-analysis evaluating EGP safety supports this showing a rate of severe adverse events between 1.6% and 3.1%, mainly consisting of pain and nausea requiring in-hospital management (~1%), gastrointestinal bleeding (~0.6%), and perigastric fluid collection (0.5%) [5]. In comparison surgical bariatric interventions report a rate of severe adverse events ranging from 0.8% to 5.6% for patients undergoing laparoscopic gastric plication (LGP) [6] and 1.4%–9.4% for those undergoing Roux-en-Y gastric bypass (RYGB) [7].

Nevertheless, a favorable safety profile does not eliminate the occurrence of important adverse effects. Thus, it is important for bariatric surgeons and other professionals involved in the care of patients with obesity to be aware of potential complications of EGP and effective strategies to solve them. Here we present two cases illustrating both early and late complications associated with EGP, requiring surgical management.

## Case 1

A 24-year-old woman with a history of bipolar disorder type II, polycystic ovary syndrome, and long-standing obesity grade I (body mass index [BMI]: 30.2 kg/m^2^), underwent EGP 2 weeks prior to presentation. On the 15^th^ day post procedure, she developed sudden-onset severe epigastric pain, visual analog scale 10/10, associated with vomiting, and diaphoresis. Self-medication with analgesics and antispasmodics did not relieve her symptoms and the patient presented to the emergency room.

On physical examination, she was tachycardic and in distress, with marked epigastric tenderness. Laboratory studies showed leukocytosis (17 K/uL) with neutrophilia (82%) and elevated C-reactive protein (10.63 mg/L). Contrast-enhanced abdominal computed tomography (CT) revealed a heterogeneous collection in the lesser sac (~500 cc), free fluid, and hyperdense areas suggestive of suture dehiscence.

Urgent diagnostic laparoscopy was performed, revealing ~700 mL hemoperitoneum and an ~800 mL retrogastric hematoma. Active bleeding from the right gastroepiploic artery was identified, along with a subserosal hematoma between gastric plications. Hemostasis was achieved using clips and harmonic devices, with evacuation of collections and adhesiolysis. Total estimated blood loss was 1500 mL. Methylene blue test confirmed gastric integrity.

Postoperatively, the patient developed acute post-hemorrhagic anemia (Hb 8.9 g/dL), requiring transfusion of two units of packed red blood cells. She progressed favorably, remained hemodynamically stable, and tolerated diet advancement. She was discharged on postoperative day 4 with oral antibiotics, analgesics, and anticoagulation.

## Case 2

A 31-year-old woman with obesity grade II, (BMI): 36.4 kg/m^2^, metabolic syndrome, metabolic dysfunction–associated steatotic liver disease, obstructive sleep apnea, and prior EGP performed 2 years ago presented with complaints of dyspepsia and recurrent weight gain. The dyspepsia started since the EGP procedure and pharmacological treatment with several regimens of proton pump inhibitors (PPIs), antispasmodics, and antiflatulent drugs has not provided clinical improvement. The recurrent weight gain started 1 year after the EGP procedure, before that adequate weight loss was observed (BMI reduced to 28.8 kg/m^2^). The patient had been adherent to a physical activity regimen of four times per week and nutritional advice. An upper gastrointestinal endoscopy was performed, revealing dehiscence of the plication. Definite treatment with RYGB. The gastric remnant contained embedded metallic cinches from the Apollo endoscopic suturing system used for the EGP; therefore, the remnant was resected and removed. After removal RYGB is confectioned without complications. The removed stomach segment is sent for histopathological analysis revealing chronic active gastritis involving the antral and corpus mucosa. Atrophy stage according to the Operative Link for Gastritis Assessment (OLGA) system was 0. The second day after the revisional intervention the patient was discharged asymptomatic except for mild pain in the surgical site relieved with oral analgesics. Prophylaxis with daily PPIs is prescribed for 1 month. In subsequent follow-up visits the patient reports complete cessation of the dyspepsia symptoms and adequate weight loss is noted.

## Discussion

Reports on EGP complications focus on early or intermediate postoperative adverse effects. Regarding those the procedure is considered to have a similar safety profile when compared to surgical interventions, particularly with LGP [8].

As it was mentioned earlier, the most common complications include pain and nausea, GI bleeding, and perigastric fluid collection [5]. Case 1 exhibits the later adverse event, but superimposed with severe, acute, life threating hemoperitoneum. Although rare in an endoscopic intervention, this is a plausible complication due to the mechanical stress imposed on the vasculature dragged with the plication.

The preferred diagnosis modality would depend on patient’s hemodynamic stability. Stable patients could benefit from contrast-enhanced angiography as this study can identify not only the bleeding vessel but also vascular anomalies that may have caused the injury in first place; unstable however require immediate surgical exploration without delay for imaging [9]. In case 1 a reasonable compromise is presented as the patient was relatively stable and contrast-enhanced abdominal CT was done before performing urgent diagnostic laparoscopy.

Long-term complications like the one described in case 2 have been less characterized. One valuable source of information is a prospective cohort study that followed 203 patients up to 5 years after procedure. It found two cases of dyspeptic symptoms like the one described here. Although a crucial difference is that in those patients surgical sutures were in place when endoscopic assessment was performed. However bridging fibrosis bands were found as well and the symptoms relieved after reversal of the procedure, suggesting a similar mechanism of action [10].

Regarding recurrent weight gain, the same study as well as a retrospective cohort analysis that followed 248 patients for up to 2 years report that adequate weight loss is generally maintained over time. With a total body weight loss mean of ~15% both for 2 and 5 years follow-up [10, 11]. Although wider ranges were observed in the 5 years follow-up with only ~60% of patients maintaining total body weight loss of 10% [10]. In the case presented here recurrent weight gain was likely a consequence of insufficient narrowing of the stomach due to suture failure. This is supported by the report of the patient’s diagnostic upper endoscopy. Since the patient was evaluated only 2 years after the original procedure, it is reasonable to hypothesize that had the plication maintained its original size total weight loss would have been on the expected 15% range.

Endoscopic treatments are emerging alternatives to bariatric surgery but lack long-term efficacy. Recognizing and maintaining a high index of suspicion for potential complications is crucial in patients undergoing these emerging procedures. This report illustrates two distinct but clinically significant complications: an early hemorrhagic event and a late suture dehiscence. Both cases highlight the importance of laparoscopic surgical intervention as a strategy to resolve such complications. Case 2 also denotes the reversal of EGP with conversion to RYGP with removal of gastric remanent as a feasible strategy to relieve GI symptoms while conserving the associate weight loss benefit of bariatric interventions. Further short- and long-term studies are needed to validate the effectiveness and safety of these emerging treatments.
